# Rational Fabrication of Copper Nanoclusters and In Vitro Study of Antioxidant Property

**DOI:** 10.3390/nano15050360

**Published:** 2025-02-26

**Authors:** Jiale Zhang, Xiao Liang, Mijia Yan, Qiuhong Zhang, Hangrong Chen

**Affiliations:** 1 School of Chemistry and Materials Science, Hangzhou Institute for Advanced Study, University of Chinese Academy of Sciences, 1 Sub-Lane Xiangshan, Hangzhou 310024, China; zhangjiale221@mails.ucas.ac.cn (J.Z.); liangxiao22@mails.ucas.ac.cn (X.L.); yanmijia22@mails.ucas.ac.cn (M.Y.); 2 State Key Laboratory of High Performance Ceramics and Superfine Microstructure, Shanghai Institute of Ceramics, Chinese Academy of Sciences, 1295 Ding-Xi Road, Shanghai 200050, China

**Keywords:** oxidative stress, nanocluster, reactive oxygen species, antioxidant activity, bioavailability

## Abstract

Oxidative stress, resulting from an imbalance between reactive oxygen species (ROS) and antioxidants, is a critical factor in the pathogenesis of a wide range of diseases. The excessive accumulation of ROS can cause severe cellular damage, leading to tissue dysfunction and disease progression. The development of nanomaterials with antioxidant properties presents a promising strategy for addressing this challenge. Herein, we report the fabrication of albumin-biomineralized copper nanoclusters (BCNCs) as a novel antioxidant platform and evaluate their effectiveness in combating oxidative stress. Our results show that BCNCs exhibit potent ROS scavenging abilities and protect cells from oxidative stress-induced damage, highlighting their potential as an effective therapeutic strategy for oxidative stress-related diseases.

## 1. Introduction

Oxidative stress mainly results from an imbalance between reactive oxygen species (ROS) and antioxidant systems, plays a pivotal role in various physiological and pathological processes [1]. ROS, including superoxide anions (·O_2−_), hydrogen peroxide (H_2_O_2_), and hydroxyl radicals (·OH), are produced during cellular metabolism, and serve as essential regulators of cellular processes, including signal transduction, gene expression, and immune responses, at controlled levels. However, excessive accumulation of ROS can overwhelm the intracellular antioxidant defense system, contributing to cellular damage [2]. Oxidative stress is implicated in the development and progression of numerous diseases [3], including liver injury [4], neurodegenerative disorders [5], cardiovascular diseases [6], kidney injury [7], and diabetes [8]. The deleterious effects of ROS on cellular components, such as lipids, proteins, and DNA, are central to the pathogenesis of these diseases. Excessive ROS accumulation at the lesion site causes cascading amplified cell damage and even cell necrosis [9].

Among the numerous conditions associated with oxidative stress, liver injury is a prime example [10]. Acetaminophen (APAP)-induced liver injury occurs as a result of excessive ROS production following APAP metabolism. APAP overdose-induced hepatotoxicity is primarily attributed to its metabolite, N-acetyl-p-benzoquinone imine (NAPQI), whose overdose depletes GSH, leading to an accumulation of reactive oxygen species (ROS). This oxidative imbalance ultimately triggers oxidative stress-mediated hepatocyte necrosis and liver injury [11]. Given the central role of oxidative stress in APAP-induced liver injury, antioxidants play a crucial role in scavenging ROS, restoring redox homeostasis, and preventing liver damage, highlighting their therapeutic potential in hepatoprotection.

Despite antioxidants with ROS scavenging properties, such as L(+)-Ascorbic acid (L-AA) and N-acetylcysteine (NAC), having been approved as therapeutic strategies for treating oxidative stress-related conditions, traditional antioxidants face significant limitations in clinical use due to poor bioavailability, low stability, and limited efficacy [12]. Bioavailability of traditional antioxidants is limited by the low absorption and the degradation during delivery, preventing sufficient therapeutic concentrations in systemic circulation [13,14,15]. Furthermore, the short half-life of traditional antioxidants, requiring frequent dosing to maintain their effects, complicates long-term therapeutic use. Traditional antioxidants also have non-negligible adverse effects, including gastrointestinal discomfort, anaphylactoid reactions, and potential hepatotoxicity at high doses [16]. Therefore, there is an urgent need to develop novel therapeutic strategies with enhanced efficiency to mitigate oxidative stress.

Nowadays, advancements in nanomedicine have significantly expanded their potential for clinical applications, particularly in the treatment of oxidative stress-related diseases [10,17,18]. Nanomaterials with intrinsic antioxidant properties have been developed to efficiently scavenge ROS and restore redox homeostasis. Several nanomedicines, such as cerium dioxide nanozymes (CeO_2_ NPs) [19], melanin-like nanoparticles (MNPs) [20], selenium nanoparticles (Se NPs) [21], and Prussian blue nanoparticles [22], have demonstrated promising therapeutic effects in alleviating oxidative stress-induced damage. The emergence of nano-antioxidants as a therapeutic strategy offers distinct advantages, including enhanced ROS-scavenging capacity, improved stability in pathological environments, and targeted delivery capabilities [12,23]. Given these benefits, nanomedicine-based antioxidant therapies have garnered attention due to t great promise as a viable approach to mitigating oxidative stress by scavenging ROS. However, there are still several limitations that hinder the therapeutic application of nanomedicine. Despite the promising ROS clearance effect, some nanomedicine offer relatively low catalytic activity, which is detrimental to the reversal of oxidative stress [24]. Moreover, some nanomaterials incorporate non-essential metal elements, such as gold (Au) nanoparticles, and the accumulation of non-essential elements in biological systems raises concerns over potential toxicity with prolonged use [25]. More importantly, the high cost and complex synthesis process of nanomedicines can impair the clinical translation [26]. Thus, it still remains a challenge to develop nanomedicines with good biocompatibility, excellent ROS scavenging ability, and simple fabrication to enable clinical applications.

Among various nanomaterials developed for oxidative stress-related disease treatments, copper-based nanomaterials have shown distinct advantages. Copper (Cu), as an essential transition metal with intrinsic redox properties, provides copper-based nanomaterials with outstanding antioxidant capabilities and superior multiple ROS scavenging efficiency [27]. More importantly, the high catalytic activity of copper-based nanomaterials further enhances the antioxidant potential, which enables the efficient degradation of ROS even at low concentrations, making the. more efficient and feasible for therapeutic use [28,29]. As an essential trace element crucial for various biological processes, the natural presence of Cu could significantly reduce potential toxicity and adverse reactions when used therapeutically [30,31]. It has been reported that copper-based nanomaterials demonstrate excellent chemical stability and biocompatibility, ensuring prolonged therapeutic effects [24,32]. Moreover, recent studies have reported that ultrasmall Cu_5.4_O nanoparticles exhibit excellent ROS scavenging properties and have been mainly investigated for the treatment of kidney injury [24], highlighting the promise of copper-based nanomaterials in combating oxidative stress-related diseases. To sum up, the advantages of scavenging multiple ROS species, high catalytic efficiency, and biocompatibility and stability position the use of copper-based nanomaterials as a promising and highly feasible strategy for the treatment of oxidative stress-related diseases, with significant potential for clinical application.

Herein, we report a bovine serum albumin-mediated biomimetic mineralization to fabricate stabilized copper nanoclusters (named BCNCs), exhibiting good biocompatibility. The prepared BCNCs possess a broad ROS scavenging spectrum, effectively targeting multiple ROS species, including ·O_2−_, ·OH, and H_2_O_2_, surpassing many other nanomaterials targeting specific ROS species in terms of antioxidant activity. This extensive ROS neutralization ability, combined with their high catalytic efficiency, allows BCNCs to exert potent antioxidant effects at low concentrations, eliminating the need for surface modifications or extra activation. More importantly, BCNCs demonstrate excellent biocompatibility and stability, which ensure their prolonged activity in biological systems, and minimizes the risk of harmful side effects. Moreover, BCNCs offer substantial advantages in terms of cost-effectiveness and scalability, and the fabrication method is straightforward and efficient, which enables large-scale production, presenting much potential for further clinical use. Our in vitro study demonstrated that BCNCs effectively elevated overall intracellular antioxidant levels and alleviated oxidative stress injury. These results highlight the potent antioxidant activity of BCNCs and their possibility for clinical application against oxidative stress-related diseases.

## 2. Materials and Methods

### 2.1. Materials

Bovine serum albumin (BSA) was supplied from BBI (Shanghai, China). Copric chloride dihydrate (CuCl_2_·2H_2_O), sodium hydroxide (NaOH), 2,2′-Azino-bis(3-ethylbenzothiazoline-6-sulfonic acid) diammonium salt (ABTS), hydrogen peroxide solution (H_2_O_2_), potassium persulfate (K_2_S_2_O_8_), and 4-Acetamidophenol (APAP) were obtained from Macklin (Shanghai, China). Ethanol anhydrous was purchased from Sinopharm (Shanghai, China). Ethylenediaminetetraacetic acid disodium salt (EDTA-2Na) and Hydrazine solution (N_2_H_4_) were brought from Aladdin (Shanghai, China). All chemicals and reagents in this study were analytical grade and used without further purification. Nitric acid (HNO_3_) was obtained from Sinopharm (Beijing, China). Standard solutions of copper (Cu) and Rhodium (Rh) were brought from the National Institute of Metrology (Beijing, China). All assay kits involved in this study were used following the manufacturer’s instructions without further purification or other special treatments. Ultrapure water (18.2 MΩ cm^−1^ at 25 °C) purified by a Milli-Q system (Merck, Darmstadt, Germany) was used throughout the experiments.

### 2.2. Methods of Characterization

TEM images were obtained using a 120 kV Transmission Electron Microscope (TEM, Talos L120C G2, Thermo Scientific, Waltham, MA, USA). Hydrodynamic size and zeta potential was acquired using Zetasizer Ultra (Malvern, Worcestershire, UK). Absorption spectra were measured with a UV–vis spectrometer with the wavelength range of 400–800 nm (Lambda 1050+, PerkinElmer, Waltham, MA, USA). Elemental valence state was detected by X-ray photoelectron spectroscopy (XPS, ESCALAB Xi+, Thermo Fisher, Waltham, MA, USA); the C 1s peak at 284.8 eV as reference was used to calibrate the binding energies. Elemental concentration was determined by inductively coupled plasma mass spectrometry (ICP-MS, NexION 2000G, PerkinElmer, USA). Samples measured by ICP-MS were digested with HNO_3_ for 12 h. Cu standard solutions with seven different concentrations was prepared by gradient dilution, and 200 ppb Rh as the internal standard. Cu concentrations were measured with He-KED mode. Absorbances at different wavelengths were monitored with a microplate reader (Multiskan SkyHigh, Thermo Scientific, Waltham, MA, USA). Cell images were obtained using fluorescence microscopy (DM IL LED, Leica, Wetzlar, Germany), and the mean fluorescence intensity of cell images were quantified by Image J (version 1.54c 6). If not specifically mentioned, all measurements were taken at room temperature.

### 2.3. Synthesis of BCNCs

BCNCs were synthesized by biomimetic mineralization. Briefly, 200 mg BSA was dissolved in 8.5 mL deionized water and stirred for 10 min (500 rpm) to ensure complete dissolution. Then, CuCl_2_·2H_2_O aqueous solution (1 mL, 100 mM) was added, and the color of solution changed from colorless to blue. After 3 min, NaOH (500 μL, 1 M) aqueous solution was added to adjust the pH to about 12 and the mixture turned a dark purple. After that, N_2_H_4_ (500 μL, 500 mM) was added and the mixture was kept for 12 h at 37 °C and the ultimate solution appeared brownish yellow in color. The above solution was purified by dialysis (MW 14 kDa) for 2 days and then stored at 4 °C. The concentrations of Cu in BCNCs were quantified by ICP-MS, and the samples were measured three times. The final concentration of BCNCs was determined based on their copper content.

### 2.4. Superoxide Anion (·O_2−_) Clearance Ability of BCNCs

The ·O_2−_ clearance ability of BCNCs was detected using a Total SOD Activity Assay Kit (Beyotime, Shanghai, China), following the kit’s instructions. Generally, WST-8 reacts with ·O_2−_ catalyzed by Xanthine Oxidase to produce a water-soluble formazan dye, and the amount of ·O_2−_ can be calculated by the WST-8 product. Absorbance at 450 nm was assayed by mixing different concentrations of BCNCs (5–20 μg/mL) with kit solutions homogeneously for 30 min at 30 °C, and the ·O_2−_ clearance ability was calculated. A sample with ultrapure water as a negative control was used to validate the results.

### 2.5. Hydroxyl Radical (·OH) Clearance Ability of BCNCs

A Hydroxyl Free Radical Scavenging Capacity Assay Kit (Solarbio, Beijing, China) was used to detect the concentration of ·OH. Briefly, 50 μL BCNCs (0.25–2 μg/mL) and 150 μL test working solution were mixed for 60 min at 30 °C, and monitored at 536 nm using a microplate reader. A sample with ultrapure water as a negative control was used to validate the results.

### 2.6. Hydrogen Peroxide (H_2_O_2_) Clearance Ability of BCNCs

The content of H_2_O_2_ was measured using Hydrogen Peroxide Assay Kit (Beyotime, China). The detection is achieved by oxidizing Fe^2+^ with H_2_O_2_ to produce Fe^3+^, which then forms a purple product with xylenol orange in a specific solution. After treating different concentrations of BCNCs (1–10 μg/mL) for 3 h at 30 °C, the solutions were ultrafiltered (Mw 30 kDa) according to the kit instructions and the H_2_O_2_ clearance ability of BCNCs was calculated. Absorbance at 450 nm was monitored at 560 nm using a microplate reader. H_2_O_2_ standard solutions with different concentrations were used to construct a standard curve.

### 2.7. Total Antioxidant Ability of BCNCs

Total antioxidant ability was determined by using ABTS as a color developer. Specifically, ABTS was oxidized to blue ABTS· in the presence of an appropriate oxidant, such as K_2_S_2_O_8_, and the production of ABTS· was inhibited in the presence of the antioxidant. Different concentrations of BCNCs (0.25–2 μg/mL) were co-incubated with a Total Antioxidant Capacity Assay Kit (Beyotime, China) for 30 min at 30 °C, and the absorbance at 734 nm was monitored using a microplate reader. A sample with ultrapure water as a negative control was used to validate the results.

### 2.8. Cell Culture

Murine hepatocyte cells (BNL CL.2, Lot number: C5532) were purchased from BDBIO Company (Hangzhou, China). The BNL CL.2 cells were cultured and maintained in Dulbecco’s Modified Eagle Medium (DMEM, Gibco, Thermo Fisher, Waltham, MA, USA) supplemented with 10% fetal bovine serum (FBS, Gibco, USA), and 1% streptomycin and penicillin (Solarbio, China) at 37 °C in a 5% CO_2_ incubator. The cells were passaged every 2 days, and cells in the logarithmic growth phase were used for further experiments. Typically, BNL CL.2 cells were cultured in 96-well plates or 6-well plates at a density of 1 × 10^5^/well for 24 h.

### 2.9. In Vitro Cytotoxicity of BCNCs

BNL CL.2 cells were seeded into 96-well plates at a density of 1 × 10^5^/well for 24 h, then the cells were incubated with different concentrations of BCNCs (0.125–5 μg/mL) in serum-free DMEM medium (Gibco, USA) for 12 h and 24 h, respectively. Subsequently, the cells were washed gently using PBS twice, and the cell viabilities were detected using a CCK-8 assay kit (Beyotime, China). The absorbance was monitored at 450 nm using a microplate reader, and the cell viabilities were calculated.

### 2.10. Hepatoprotective Effect of BCNCs

To mimic liver injury, BNL CL.2 cells were exposed to 6 mM acetaminophen (APAP) along with a series of BCNC concentrations (0.125–5 μg/mL) in serum-free DMEM (Gibco, USA) for 12 h. Then, the culture medium was discarded, and the cells were washed gently using PBS, before cell viability was assayed using a CCK-8 assay kit (Beyotime, China) to verify the hepatoprotective effect of BCNCs.

### 2.11. Cellular Uptake

Considering the fluorescent properties of BCNCs, cellular uptake can be determined by the fluorescence of cells after incubation with BCNCs. In detail, BCNCs diluted by serum-free DMEM medium (Gibco, USA) to 1 μg/mL were incubated with BNL CL.2 cells. After 12 h, the culture medium containing BCNCs was discarded, and the adherent cells were washed with 10 mM EDTA-2 Na to remove the excess BCNCs, then washed again with PBS. The cell fluorescence was observed with emission at 365 nm.

### 2.12. Intracellular ROS Scavenging Effect

To estimate the intracellular ROS scavenging ability of BCNCs, BNL CL.2 cells were simultaneously treated with 6 mM APAP and 1 μg/mL BCNCs in serum-free DMEM (Gibco, USA) for 12 h. The level of intracellular ROS was detected using 2′,7′-dichlorodihydro-fluorescein diacetate (DCFH-DA, Beyotime, China). Typically, cells in 6-well plates were stained with DCFH-DA for 20 min in the dark. The fluorescence images were captured using a fluorescence microscope, and quantified using Image J.

### 2.13. Enzyme-Linked Immunosorbent Assay (ELISA)

BNL CL.2 cells were seeded in 6-well plates at a density of 1 × 10^5^/well for 24 h, then incubated with BCNCs (1 μg/mL) for 12 h. The intracellular level of glutathione (GSH) and Malondialdehyde (MDA) were detected using a Reduced GSH assay kit (A006-2, Nanjing Jiancheng, Nanjing, China) and MDA assay kit (A003-1, Nanjing Jiancheng, China), respectively. BCA contents of sample cells were quantified using a BCA protein quantification kit (BF0026, Boerfu, Wuhan, China).

### 2.14. Statistical Analysis

Data are shown as the mean ± standard error of the mean (SEM). Significant differences were analyzed with GraphPad (Prism 8, San Diego, CA, USA). Differences in data in multiple experimental groups were analyzed by one-way analysis of variance (ANOVA); Tukey’s post hoc test was used after ANOVA to determine pairwise significance. Each experiment was repeated three times, with duplicate measurements for each replicate. The *p* value < 0.05 was considered significant difference.

## 3. Results

**Figure 1:**
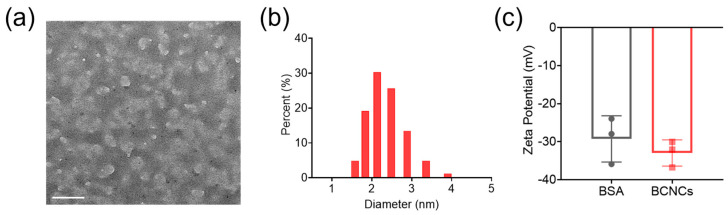
(**a**) TEM image of BCNCs, scale bar: 100 nm. (**b**) DLS results of BCNCs assembled in aqueous solution. (**c**) Zeta potential of BSA and BCNCs in aqueous solution. n = 3 samples in c; data are presented as mean ± SEM.

The BCNCs were synthesized using a one-pot biomineralization process. Firstly, BCNCs were characterized by TEM and found to be uniformly distributed in BSA (white) in the form of nanoclusters (black) of around 1.5–2 nm (Figure 1a), which is consistent with the hydrodynamic size (2.14 nm, PI: 0.258) measured by dynamic light scattering (DLS, Figure 1b). The zeta potential of BCNCs was measured to be −32.96 ± 4.17 mV, close to that of BSA, indicating that BCNCs were well dispersed in solution and successfully synthesized using BSA as a biomimetic template (Figure 1c).

**Figure 2:**
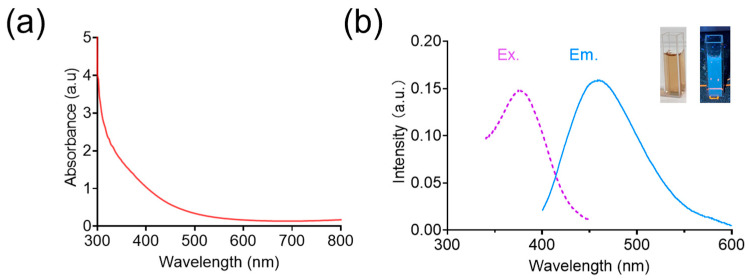
(**a**) UV–vis absorption spectra of BCNCs. (**b**) Fluorescence excitation and emission spectra of BCNCs.

The UV–vis absorption of BCNCs showed no significant absorption peaks in the wavelength range of 400–800 nm (Figure 2a), which could be ascribed to the relatively homogeneous small-sized nanoclusters of BCNCs and the low abundance of larger nanoparticles in the medium, and similar results can also be found in previous reports [33,34]. Moreover, the optical properties of BCNCs were detected using fluorescence spectroscopy. As shown in Figure 2b, the BCNCs displayed a characteristic fluorescence emission at 460 nm under an excitation wavelength of 375 nm, demonstrating successful synthesis of the nanoclusters [35].

**Figure 3:**
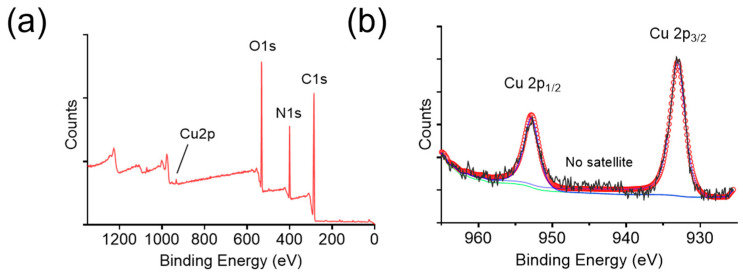
(**a**) XPS full-scan spectrum of BCNCs. (**b**) High-resolution XPS spectrum of the Cu 2p of BCNCs (black: spectrum, blue: Cu 2p scan, green: background, red: fitting curve).

Furthermore, the elemental composition of BCNCs was analyzed using XPS. The C 1s peak at a binding energy of 284.8 eV was used as a reference for surface charge calibration. The full scan spectrum revealed the presence of C, N, O, and Cu (Figure 3a), therein C, N, and O originating from the BSA protein. To determine the oxidation state of copper, high-resolution XPS spectra of Cu 2p were acquired (Figure 3b). The Cu 2p_3/2_ and 2p_1/2_ binding energies were located at 933.04 and 952.81 eV, respectively, implying the presence of Cu^0^. Nevertheless, the Cu^0^ 2p_3/2_ binding energy differs from that of Cu^+^ by only ~0.1 eV, making it difficult to exclude the presence of Cu⁺ [36]. Notably, the absence of satellite features in Figure 3b indicates that Cu^2+^ is virtually absent in BCNCs, since Cu^2+^ typically exhibits characteristic satellite peaks at ~943 eV [37]. Overall, the abundant presence of reduced-state copper ions in BCNCs highlights their potential for ROS scavenging and oxidative stress mitigation.

**Figure 4:**
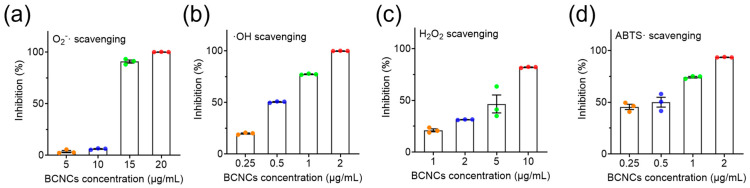
(**a**) The ·O_2−_ scavenging ability of BCNCs. (**b**) The ·OH scavenging ability of BCNCs. (**c**) The H_2_O_2_ scavenging ability of BCNCs. (**d**) Total antioxidant ability of BCNCs. n = 3 samples in (**a**–**d**); data are presented as mean ± SEM.

To verify the antioxidant capacity of BCNCs, three representative ROS, ·O_2−_, ·OH, and H_2_O_2_, were selected for scavenging experiments [38]. As shown in Figure 4a, BCNCs effectively eliminated nearly 100% of ·O_2−_ at a concentration of 15 μg/mL. Additionally, at 10 μg/mL, approximately 84% of H_2_O_2_ was decomposed (Figure 4c). It is worth mentioning that even at an extremely low concentration of 2 μg/mL, BCNCs achieved complete ·OH scavenging and exhibited superior total antioxidant capacity (Figure 4b,d). All the above results validate the excellent efficiency of BCNCs in scavenging broad-spectrum ROS and underscore their significant potential in mitigating oxidative stress-induced damage in vivo.

**Figure 5:**
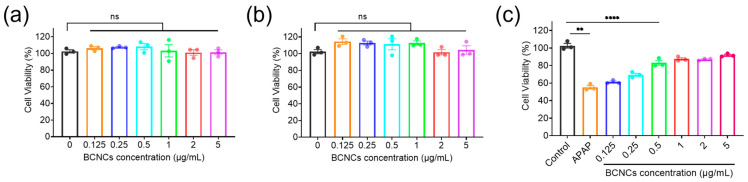
(**a**) Cell viability of BNL CL.2 cells treated with BCNCs for 12 h. (**b**) Cell viability of BNL CL.2 cells treated with BCNCs for 24 h. (**c**) Hepatoprotective effect of BCNCs. n = 3 samples in (**a**–**c**); data are presented as mean ± SEM, with ns indicating no significance; ** indicating *p* < 0.01; **** indicating *p* < 0.0001.

Encouraged by the superior antioxidant capacity of BCNCs in vitro, we further investigated the protective effects of BCNCs. Liver injury, particularly acetaminophen (APAP)-induced hepatotoxicity, serves as a classic example of oxidative stress-induced damage [4]. APAP metabolism generates reactive metabolites that deplete cellular antioxidants, leading to the accumulation of ROS and subsequent hepatocyte damage [39]. Therefore, APAP-induced hepatocyte injury was established to evaluate the antioxidative potential and therapeutic role of BCNCs at the cellular level. First, the cytotoxicity of BCNCs was evaluated using the CCK-8 assay. The results demonstrated that BCNCs exhibited negligible cytotoxicity within the tested concentration range, as BNL CL.2 cells maintained excellent viability after 12 or 24 h of co-incubation with BCNCs at concentrations up to 5 μg/mL (Figure 5a,b). These findings indicate that BCNCs possess favorable biocompatibility and low toxicity. Excessive APAP exposure is known to induce oxidative stress in hepatocytes, leading to excessive ROS generation and subsequent cellular damage. To assess the hepatoprotective effects of BCNCs at the cellular level, an APAP-induced hepatocyte injury model was established using BNL CL.2 cells. As shown in Figure 5b, treatment with 6 mM APAP resulted in significant cytotoxicity, with cell death rates exceeding 40%. Notably, even at an extremely low concentration of 0.5 μg/mL, BCNCs effectively rescued APAP-induced cell damage, highlighting their potential in mitigating oxidative stress-related hepatocyte injury.

**Figure 6:**
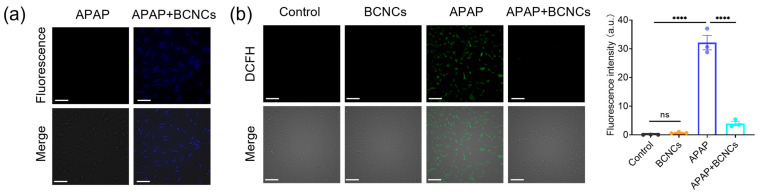
(**a**) Cellular uptake of BCNCs, scale bar: 50 μm. (**b**) Fluorescence images of BCNCs scavenging intracellular ROS; the mean fluorescence intensities of images were quantified by Image J. Scale bar: 50 μm. n = 3 samples in (**b**); data are presented as mean ± SEM, with ns indicating no significance; **** indicating *p* < 0.0001.

Furthermore, the fluorescence property and low toxicity of BCNCs made it possible to test the cellular uptake by cell labeling. Under UV light excitation, blue fluorescence could be observed in the BNL CL.2 cells, which is the characteristic fluorescent color of BCNCs (Figure 6a), demonstrating the successful uptake of BCNCs by the cells and their ability to exert antioxidant effects within cells. Next, the ability of BCNCs to protect cells from ROS damage was tested using the DCFH-DA assay. Upon entry into the cell, DCFH is oxidized by intracellular ROS, generating green fluorescence. Compared to the control group of untreated BNL CL.2 cells, cells treated with BCNCs did not trigger a visible change in green fluorescence, which clearly indicated that BCNCs themselves do not cause intracellular ROS production. Severe oxidative stress in liver injury is induced by excessive ROS production [10]. Following APAP treatment, a significant enhancement in green fluorescence was observed, indicating a dramatic increase in the intracellular ROS levels and exacerbation of oxidative stress-induced damage. Notably the level of ROS was obviously reduced after co-incubation with BCNCs, confirming that BCNCs possess the ability to alleviate intracellular oxidative stress (Figure 6b).

**Figure 7:**
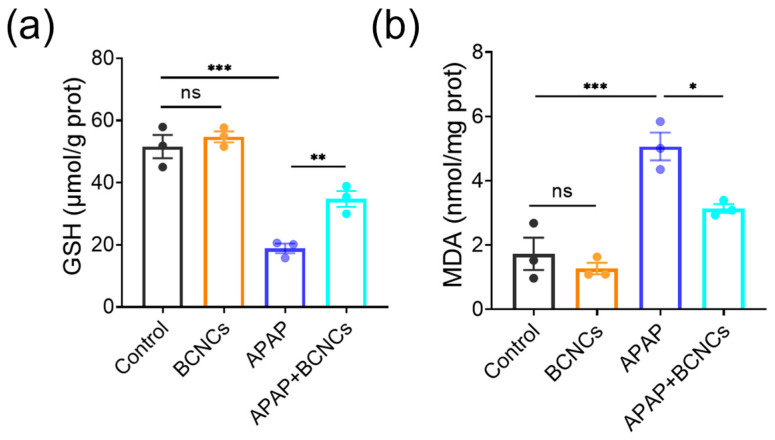
(**a**) Intracellular GSH level of BNL CL.2 cells after different treatments. (**b**) Intracellular MDA level of BNL CL.2 cells after different treatments. n = 3 samples in (**a**,**b**); data are presented as mean ± SEM, with ns indicating no significance; * indicating *p* < 0.05; ** indicating *p* < 0.01; *** indicating *p* < 0.001.

In addition, intracellular indicators related to oxidative stress were quantified using ELISA to explore the possible mechanism of BCNCs on hepatoprotection. GSH is one of the most commonly used non-enzymatic indicators of intracellular antioxidant substances [40], and the expression of GSH in APAP-treated cells was significantly reduced, and the BCNCs altered this trend (Figure 7a), which reflected that the addition of BCNCs helped to restore the overall antioxidant performance. MDA is a major product of lipid peroxidation injury and is considered a biomarker closely related to oxidative stress [41]; thus, the degree of cellular oxidative stress can be assessed by MDA content. As shown in Figure 7b, the MDA level of APAP-treated cells increased substantially, but levels in the damaged cells approached normal levels after BCNC incubation. According to the above results, it can be supposed that the APAP-treated group exhibits increased oxidative stress damage, while BCNCs can scavenge the generated intracellular ROS and rebound overall antioxidant properties, thus in turn reducing the level of cellular oxidative stress. Briefly, it can be inferred that BCNCs are expected to show promise in the treatment of APAP-induced liver injury.

## 4. Discussion and Conclusions

It has been recognized that excessive ROS is involved in the pathogenesis of various oxidative stress-related diseases, and nanomaterials with intrinsic antioxidant properties hold great promise for scavenging ROS and mitigating the progression of oxidative damage [12]. Previous studies have demonstrated the significant antioxidant potential of Cu-based nanomaterials. For instance, Cu NPs have been shown to catalytically scavenge H_2_O_2_ in biological sensing applications [42], while Cuprous oxide (Cu_2_O) NPs possess activity to deactivate H_2_O_2_ and ·OH through the electron transfer reaction [43]. Given the complexity of ROS profiles in oxidative stress environments, there is a strong demand for nanomedicines capable of broad-spectrum ROS scavenging [24].

In this study, novel Cu-based nanoclusters, i.e., BCNCs, were synthesized, which exhibited a uniform ultrasmall size of approximately 1.5–2 nm and demonstrated excellent stability. Under 375 nm excitation, they display a characteristic fluorescence emission peak at 460 nm, confirming the fluorescence properties of nanoclusters. Moreover, the prepared BCNCs have been verified to possess promising reducing properties and exhibit superior broad-spectrum ROS scavenging activity, including ·O_2−_, ·OH, and H_2_O_2_. Notably, the high catalytic efficiency of BCNCs ensures potent antioxidant effects even at low concentrations. Additionally, the excellent biocompatibility of BCNCs has been validated at the cellular level, ensuring long-term activity in organisms while significantly minimizing the risk of toxicity and side effects.

To evaluate their therapeutic potential, we employed an acetaminophen (APAP)-induced liver injury model, which confirmed the ability of BCNCs to protect hepatocytes from oxidative damage. Notably, compared to other nanomedicine-based hepatoprotective strategies, such as CeO_2_ NPs (5 mM) [19] and Se NPs (20 μg/mL) [44], BCNCs exhibited superior efficacy at an extremely low concentration (1 μg/mL), significantly enhancing intracellular antioxidant defenses and mitigating oxidative stress-induced injury. These results support BCNCs as a promising nanomedicine for combating oxidative stress-induced injury and warrant further investigation in vivo to explore their clinical applicability.

In summary, we herein successfully synthesized albumin-biomineralized copper nanoclusters (BCNCs) and demonstrated their potent antioxidant properties of mitigating oxidative stress in vitro. The prepared BCNCs exhibited remarkable multiple ROS scavenging abilities and hold great efficiency for elevating the overall intracellular antioxidant level and protecting cells from oxidative stress damage induced by excessive ROS accumulation. Moreover, BCNCs possess excellent antioxidant effects even at low concentrations, reducing their systemic exposure and limiting copper accumulation in non-target organs, which would ensure therapeutic efficacy while minimizing toxicity, exhibiting them as a promising candidate for clinical application in the treatment of oxidative stress-related diseases. Future studies will focus on optimizing the in vivo stability, bioavailability, and therapeutic efficacy of BCNCs to realize their full potential in clinical applications for oxidative stress-related conditions, including neurodegenerative disorders, cardiovascular diseases, and kidney injury. Meanwhile, incorporating other metal elements or combining copper with organic or inorganic compounds may improve their stability and reduce the toxicity risks while maintaining or enhancing their antioxidant properties. Furthermore, further functionalization of copper-based nanoclusters for targeted drug delivery and disease diagnostics holds significant promise for future advancements. With further refinement and comprehensive in vivo evaluations, BCNCs could become a safe and effective therapeutic strategy for a wide range of oxidative stress-related disorders. Overall, this study provides a possibility for the development of copper-nanoclusters as a novel class of antioxidants, offering a promising avenue for combating oxidative stress in various clinical settings.

## Data Availability

The data presented in this study are available in the manuscript.

## Abbreviations

The following abbreviations are used in this manuscript:

APAP	Acetaminophen
GSH	Glutathione
ROS	Reactive oxygen species
BSA	Bovine serum albumin
MDA	Malondialdehyde
